# Quality of Life Measures in Advanced Endometrial Cancer: A Systematic Review of Reporting Practices in Phase III Clinical Trials

**DOI:** 10.3390/cancers18020258

**Published:** 2026-01-14

**Authors:** Justine Himpe, Marjolein Orije, Emiel A. De Jaeghere, Katrien Vandecasteele, Hannelore Denys

**Affiliations:** 1 Department of Medical Oncology, Ghent University Hospital, 9000 Ghent, Belgium; 2 Cancer Research Institute Ghent (CRIG), 9000 Ghent, Belgium; 3 Gynecological Pelvic Oncology Network (GYPON), 9000 Ghent, Belgium; 4 Department of Radiation Oncology, Ghent University Hospital, 9000 Ghent, Belgium

**Keywords:** endometrial cancer, quality of life, chemotherapy, immunotherapy, targeted therapy

## Abstract

**Background:** Advanced endometrial cancer is associated with poor survival. With the advent of molecular classification and novel systemic therapies—including immunotherapy and targeted agents—treatment regimens have become increasingly complex. While these approaches aim to improve survival, they also potentially introduce long-term toxicities and treatment burden, reinforcing the importance of incorporating health-related quality of life (HRQoL) and patient-reported outcomes (PROs) into clinical trials. **Methods:** A systematic review was conducted of phase III randomized controlled trials (RCTs) in advanced, recurrent, or metastatic endometrial cancer evaluating systemic treatment registered on ClinicalTrials.gov and published up to 30 November 2025. Extracted data included study characteristics, HRQoL instruments, reporting formats, adherence to CONSORT-PRO, and timing of HRQoL dissemination (relative to primary efficacy reports). **Results:** Eight phase III RCTs published between 2020 and 2024 were included. Although HRQoL was consistently designated as a secondary endpoint, reporting within pivotal efficacy publications was limited. Most reports presented mean changes from baseline using the EORTC QLQ-C30, QLQ-EN24, and EQ-5D-5L. None of the primary reports reported time-to-deterioration analyses or the proportions of patients improving/deteriorating. Adherence to CONSORT-PRO was low, with only a minority of items addressed. Dedicated QoL publications were delayed by up to 25 months after primary efficacy reports and typically appeared in journals with lower impact factors. **Conclusions:** Despite routine inclusion of HRQoL measures in trial protocols, reporting remains inconsistent, limited in scope, and often delayed. Strengthening adherence to established frameworks is essential to ensure that HRQoL endpoints are predefined, analytically robust, and disseminated alongside efficacy data—particularly in a rapidly evolving therapeutic landscape.

## Simple Summary

Recent progress in the management of advanced endometrial cancer, driven by molecular classification and the emergence of novel systemic therapies such as immunotherapy and targeted agents, has transformed the therapeutic landscape. Although these approaches aim to prolong survival, they can also lead to cumulative side effects and a considerable treatment burden. This highlights the importance of incorporating health-related quality of life (HRQoL) measures into clinical research. However, the collection and reporting of quality of life data in advanced endometrial cancer studies are often inconsistent, incomplete, or delayed, reducing their relevance for patient-centered decision-making. Timely, transparent, and methodologically robust evaluation and reporting of HRQoL outcomes are essential to contextualize clinical benefits, facilitate shared decision-making, and balance survival benefits with their real-world impact on patients’ daily lives in a rapidly evolving therapeutic landscape.

## 1. Introduction

Endometrial cancer is the sixth most common cancer in women and the most prevalent gynecological cancer in high-income countries [1,2]. Obesity and advancing age are important risk factors contributing to the rising incidence of endometrial cancer, while Lynch syndrome confers a hereditary predisposition to the disease [1,3,4,5]. Approximately two-thirds of patients present with early-stage disease, for whom surgery with risk-stratified adjuvant therapy (radiotherapy and/or chemotherapy), yields excellent outcomes [1]. In contrast, advanced, recurrent or metastatic disease has a poor prognosis with a historical median overall survival (OS) of 37 months with first-line chemotherapy (typically a platinum doublet) [6].

Beginning with the seminal paper of the molecular classification by The Cancer Genome Atlas (TCGA) in 2013, the treatment landscape has evolved significantly [7]. More personalized treatment regimens increasingly incorporate immunotherapy and targeted therapy, administered concurrently with chemotherapy and/or as maintenance therapy. These novel approaches aim to improve response rates, progression-free survival (PFS) and OS but may also introduce acute and/or long-term toxicities, treatment burden, and financial implications.

As treatment regimens expand to triplet therapies (chemo doublet with immunotherapy) and quadruplet therapies (chemo doublet with immunotherapy and PARP-inhibition), it is essential to carefully evaluate their impact on health-related quality of life (HRQoL) to contextualize the potential antitumor benefits. In addition, patients may also suffer from the burden of treatment when therapies are extended to 2 or 3 years of maintenance therapy. Furthermore, patients may experience HRQoL impairment not only from advanced disease but also from early-stage treatments, such as lymphoedema and polyneuropathy, as well as bladder, bowel, and sexual dysfunction [8].

HRQoL is a multidimensional concept of the impact of disease and treatment on physical, psychological, and social aspects of functioning and well-being [9,10,11]. HRQoL measures and patient-reported outcomes (PROs) are designed to capture HRQoL experiences. PROs are outcomes reported by the patients, without interpretation by observers or clinicians [12]. It is well established that healthcare practitioners often under-detect or underestimate symptoms and their severity, particularly non-life-threatening adverse effects, which can nonetheless substantially affect HRQoL, especially during prolonged maintenance therapies [10,13,14].

Certain tools have been developed to support qualitative research on HRQoL and PROs. The SPIRIT-PRO guidelines provide recommendations for items that should be included in clinical trial protocols, while the CONSORT-PRO extension aims to improve the quality of reporting PRO data. Both are designed to ensure that high-quality data can be generated and used to inform patient-centered care [10,15,16]. However, PROs remain underreported and underused, limiting interpretability of trial findings [10]. This review systematically evaluates HRQoL and PRO reporting in landmark phase III trials for advanced, recurrent or metastatic endometrial cancer.

## 2. Materials and Methods

### 2.1. Identification of Reports

**Figure 1:**
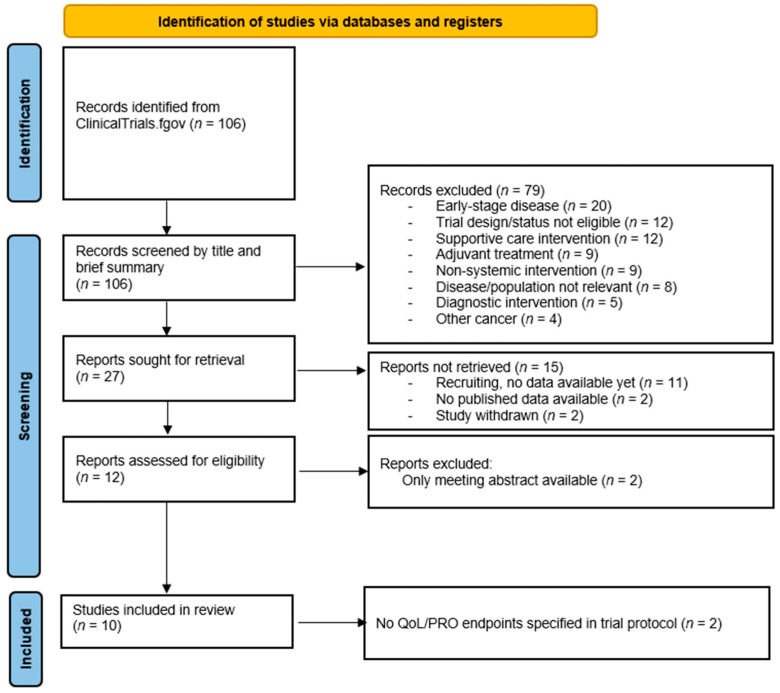
Search strategy following PRISMA guidelines.

We conducted a systematic search of the ClinicalTrials.gov database to identify phase III randomized controlled trials (RCTs) investigating systemic treatments for endometrial cancer. The search term ‘endometrial neoplasms’ was used, with filters applied to select phase III interventional studies. The search encompassed all records available through 30 November 2025, with no restriction on the earliest date. The full search process is illustrated in the PRISMA flowchart (Figure 1).

Studies were eligible for inclusion if they met the following predefined criteria: randomized phase III trials enrolling adult patients with advanced, recurrent, or metastatic endometrial cancer, evaluating any form of systemic therapy, including chemotherapy, targeted therapy, immunotherapy and/or hormonal therapy, both as single agent and combination therapy.

Studies were excluded based on the title and abstract if they (1) evaluated non-systemic interventions (e.g., radiotherapy, surgery, supportive care), (2) focused exclusively on (neo-)adjuvant treatment, (3) enrolled patients with early-stage disease (stage I and II) or (4) investigated cancers other than endometrial carcinoma.

Subsequently, for all eligible trials primary publications (i.e., first publication of trial results) were identified via supplementary searches in PubMed and Google Scholar that combined the trial name, investigational product, and the term endometrial cancer. Studies without an available full text publication were excluded. Protocols of the remaining trials were then reviewed for the inclusion of HRQoL or PRO endpoints, as these were required for further analysis.

For every included trial, subsequent reports dedicated to QoL and/or patient-reported outcomes were searched in PubMed using follow search terms: the name of the drug(s) and/or tumor type and/or the name of authors of the primary publication and/or the trial acronym/code, when present. Furthermore, conference data was sought on the websites of the following associations using the same search terms: European Society for Medical Oncology (ESMO), American Society of Clinical Oncology (ASCO), Society of Gynecologic Oncology (SGO) and European Society of Gynaecological Oncology (ESGO), International Gynecologic Cancer Society (IGCS).

This systematic review was registered in the PROSPERO registry under number 1085989.

### 2.2. Data Extraction

General trial characteristics, including the first author, trial title, publication year, and publishing journal, together with information on the used HRQoL instrument(s) and their format (e.g., mean scores at various time points, mean changes from baseline, proportions of patients responding or deteriorating, and time to deterioration), were collected by JH when available and independently reviewed by MO when available.

The extent of QoL reporting in each primary report was quantified by calculating the proportion of the results section devoted to QoL or PRO content (using Microsoft Word to calculate word count), expressed as a percentage of the total word count. Mentions of ongoing analyses were included.

During data extraction, each report’s adherence to the CONSORT-PRO extension criteria was assessed. In randomized controlled trials, where PROs are primary or important secondary endpoints, CONSORT-PRO recommends the reporting of the following five items: (1) identification of the PRO in the abstract as a primary or secondary outcome; (2) specification of a PRO hypothesis; (3) evidence of PRO instrument validity and reliability and a description of the method of data collection (analyzed as two separate items in this systematic study); (4) the statistical approach used to address missing PRO data; and (5) a discussion of the generalizability of PRO findings [10]. In accordance with CONSORT-PRO guidelines, the primary report for data extraction was prioritized. Given infrequent PRO reporting in these primary reports, trial protocols were reviewed as supplementary sources of information.

The manuscript adheres to the Preferred Reporting Items for Systematic reviews and Meta-Analyses (PRISMA) reporting guidelines. The PRISMA checklist is included in the Supplementary Materials (Table S1).

### 2.3. Statistical Analysis

Descriptive analyses were conducted in SPSS Statistics, v29.

## 3. Results

### 3.1. Characteristics of the Trials

A total of 12 trials were assessed for eligibility (Figure 1). Two were excluded due to the absence of a full-text publication. Another two trials did not pre-specify any QoL or PRO endpoints in their protocols and were therefore excluded from the final analysis, as the evaluation of PROs was a prerequisite for inclusion [17,18].

A total of eight trials were included in the analysis, with publication dates ranging from 2020 to 2024 (Table 1). Seven of the trials were industry-sponsored, while the GOG-0209 was classified as non-profit. Six trials evaluated first-line treatment strategies, one investigated maintenance therapy following first-line chemotherapy, and one focused on a second-line treatment regimen. Among the six first-line trials, four assessed combinations of chemotherapy and immune checkpoint inhibitors of which one also incorporated a PARP inhibitor during the maintenance phase. One trial evaluated chemotherapy alone, and another examined a combination of an immune checkpoint inhibitor with an anti-VEGF tyrosine kinase inhibitor. All but one trial (GOG-0209) included a maintenance or prolonged treatment strategy with a variable duration of treatment.

Regarding primary endpoints, five trials evaluated OS, either as sole endpoint or as a co-primary endpoint, while three assessed PFS. In all trials, QoL was a secondary endpoint.

### 3.2. HRQoL and PRO Reporting

**Figure 2:**
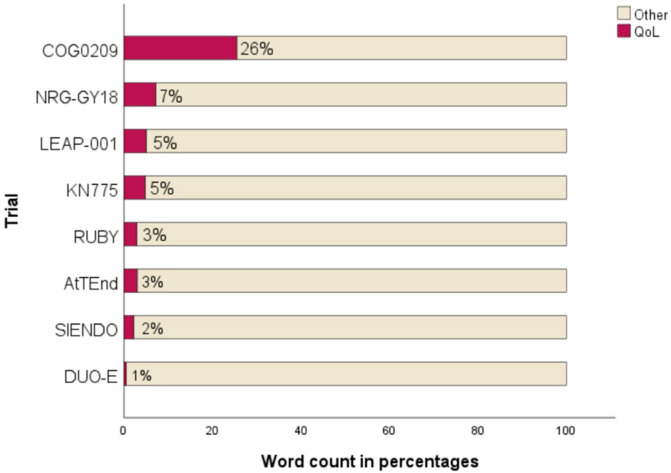
Extent of QoL reporting in results sections [6,19,20,21,22,23,24,25].

All trials with OS as primary endpoint included QoL data in the primary publication. In contrast, QoL data were not available at the time of initial publication for two of the three trials that listed PFS as the primary endpoint. The academic GOG-0209 had the highest relative word count within the results section on QoL or PRO reporting (26%) whereas the others allocated 7% or less (Figure 2).

Five trials (RUBY, AtTEnd, LEAP-001, SIENDO, KEYNOTE-775) reported mean changes from baseline, while the GOG0209 presented mean scores at multiple time points. Notably, none of the trials reported the proportion of patients experiencing improvement or deterioration, nor did any present time-to-deterioration analyses of QoL in either the primary publication or supplementary materials [20,22,23,24,25]. However, review of the trial protocols revealed that more comprehensive statistical analyses had been prespecified (Table 2). Two protocols included time-to-deterioration as a planned endpoint. The most frequently specified QoL endpoint was mean change from baseline in the global health status/QoL score of the EORTC QLQ-C30. Two trials, DUO-E and NRG-GY018, reported that analyses are ongoing and did not specify in their primary publication how PRO/QoL results will be presented.

### 3.3. HRQoL and PRO Measurement Tools

A range of HRQoL and PRO instruments were used in the trials. Endometrial cancer–specific measures comprised the EORTC QLQ-EN24 and the FACT-En-TOI. Generic or cancer-wide instruments included the EORTC QLQ-C30, EQ-5D-5L, and the FACT. Additional PRO measures evaluated were PROMIS (Patient-Reported Outcomes Measurement Information System), developed by the National Institutes of Health (NIH), and the Patient Global Impression of Severity (PGIS), a single-item measure of perceived disease or symptom severity. Symptom- and adverse event assessments were also reviewed, including the FACT/GOG–Neurotoxicity 4 and the PRO-CTCAE.

The most frequently used instruments were the EORTC QLQ-C30, the EORTC QLQ-EN24, and the EQ-5D-5L, each utilized in six trials (Table 3). Less commonly used tools are listed in Table 3. The EORTC QLQ-EN24 and FACT-En-TOI are used in 6 and 2 trials, respectively. Two trials used the FACT/GOG-Neurotoxicity 4 and the DUO-E trial uses PRO-CTCAE.

The shortest follow-up period (Table 4) was observed in the chemotherapy-only trial, lasting 26 weeks, approximately 1–2 months after the final treatment dose, assuming the maximum number of cycles was administered. The DUO-E study extended QoL assessments until the time of second disease progression. Similarly, the AtTEnd trial planned QoL follow-up either until second progression or for up to one year, whichever occurred first. In the NRG-GY018 trial, the treatment regimen included up to 14 maintenance cycles administered every 6 weeks, following an 18 weeks of combined chemotherapy and immunotherapy treatment phase. This results in a maximum treatment duration of approximately 102 weeks. However, QoL assessments in this trial concluded at week 54, well before the end of potential treatment. Other studies assessed QoL both during treatment and at predefined timepoints following treatment completion or first disease progression.

The intervals between follow-up assessments varied across trials, ranging from every 3 weeks to every 12 weeks. All trials included baseline QoL measurements prior to treatment initiation. In the DUO-E, RUBY, LEAP-001, and KEYNOTE-775 trials, follow-ups were scheduled every 3 to 4 weeks, aligning with each immunotherapy treatment cycle. These trials conducted the first follow-up at the time of the second treatment dose. In contrast, the GOG-0209 and NRG-GY018 trials scheduled the first QoL follow-up at week 6, coinciding with the third treatment dose. The AtTEnd and SIENDO trials planned their first follow-up assessments at weeks 9 and 12, respectively.

### 3.4. Quality of HRQoL Reporting According to CONSORT-PRO Extension Criteria

Overall adherence to the CONSORT-PRO extension criteria was limited (Table 5). The GOG0209 trial reported two items (items 1 and 5). The NRG-GY018 trial reported item 3a, while the LEAP-001 and SIENDO trial each reported item 4. The remaining five studies did not report any CONSORT-PRO items in their primary publications, including supplementary materials (with the exception of the trial protocol if included in the supplement).

All trials except GOG0209 and KEYNOTE-775 had protocols developed after the publication of SPIRIT-PRO (2018) and CONSORT-PRO (2013) [10,15]. Across protocols, more items were addressed than in the corresponding primary reports. Specifically, two trials (GOG0209 and NRG-GY018) included a clear PRO hypothesis; three provided evidence of PRO instrument validity; six described methods of data collection; and five report approaches for handling missing data. Item 1 (PRO identification in the abstract) was not applicable for protocol evaluation.

### 3.5. Secondary Reports on Quality of Life

**Figure 3:**
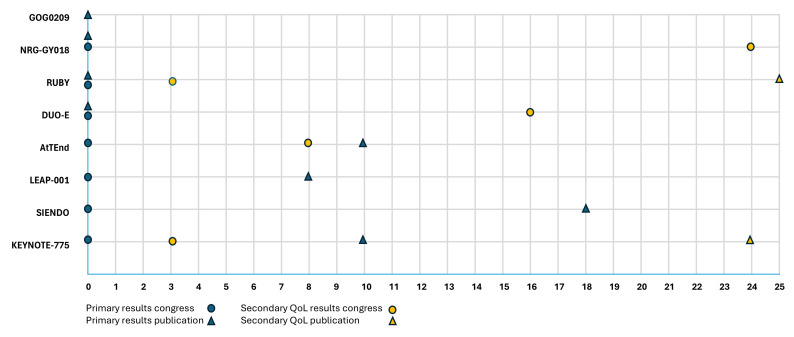
Reporting timeline of primary outcomes and quality of life results in clinical trials (months from initial presentation) [6,19,20,21,22,23,24,25].

Figure 3 shows the reporting timeline of primary efficacy outcomes and the first reporting of dedicated secondary QoL analyses. Primary results were consistently presented first at scientific congresses, followed by subsequent publication. In 3 out of 8 studies, the primary publication appeared at the time of first congress presentation, whereas the other four studies showed delays of 8, 10, or 18 months between presentation and publication. In contrast, secondary QoL outcomes were typically reported with longer delays, ranging from 3 to 24 months for the first congress report.

All primary efficacy results were published in high-impact journals, including *Journal of Clinical Oncology*, *Lancet Oncology*, and *The New England Journal of Medicine*, each consistently ranking in the top ten of their respective categories according to the 2024 Clarivate Journal Citation Reports. To date, two studies have produced dedicated QoL publications, appearing in the *International Journal of Gynecological Cancer* and the *European Journal of Cancer*, which are ranked 77th and 45th, respectively, in the Oncology category. These QoL publications were delayed by 25 months for the RUBY trial and 24 months for the KEYNOTE-775 trial compared to the first report on the efficacy results. As shown in Table 6, all primary efficacy findings were presented orally, while QoL results were presented orally in four trials and as a poster in one trial.

Overall, Figure 3 and Table 6 highlight variability in the timing and mode of dissemination of QoL findings compared with primary endpoints.

## 4. Discussion

This systematic review assessed whether HRQoL is genuinely prioritized in clinical trials of advanced, recurrent or metastatic endometrial cancer. While QoL questionnaires are frequently included in trial protocols, our findings reveal that reporting of QoL outcomes in pivotal publications remains inconsistent and limited in scope.

Review of study protocols revealed substantial inconsistencies between planned and reported secondary endpoints (and their analyses). Several trials outlined multiple QoL-related secondary endpoints, yet only a subset appeared in the primary publication, with little transparency regarding selection decisions. Explanations may include publication delays, space constraints, or reluctance to report negative or inconclusive QoL results, an issue of particular relevance where therapeutic benefit is modest. Conversely, only a subset of the collected HRQoL instruments are utilized as secondary endpoints. Together, these observations highlight the need for greater transparency of the intended role of each PRO measure in trial design and reporting.

Trials used a wide range of HRQoL and PRO tools, with heterogeneous assessment schedules and endpoints. Most analyses relied on group-level averages, such as mean difference at specified timepoints or mean change from baseline, which can be more difficult to translate into real-world individual patient care. A recent policy review by the Common Sense Oncology (CSO) initiative and the European Organisation for Research and Treatment of Cancer (EORTC) advocates for responder-based analyses that report the proportion of patients experiencing clinically meaningful changes in QoL and the duration of QoL response. Such metrics more directly adress real-world questions, namely, the likelihood that a treatment will improve a patient’s well-being and how long that improvement may last [40].

Time points for QoL assessments also differed substantially. Some trials collected QoL data until second disease progression, whereas others stopped during ongoing maintenance therapy, missing a critical window in which long-term toxicities and treatment burden are most likely to accumulate. Harmonizing QoL instruments, timing, and reporting would enhance comparability across trials and enable future, robust meta-analyses.

Ensuring access to high-quality, interpretable data, requires rigorous methodology [12,41,42]. A hypothesis-driven approach is particularly important but remains underutilized: only two of eight trials articulated an a priori QoL hypothesis, and none reported it in the primary report. Equally essential are prespecified plans for PRO analyses, including strategies for handling missing data. While all reviewed trial protocols contained a statistical analysis plan for QoL and five addressed missing data, the absence of clearly defined QoL hypotheses limits interpretability and clinical relevance of the findings [43].

Delays in QoL dissemination further highlight its limited prioritization. In most trials, QoL outcomes were presented at congresses or published separately as secondary papers of lower visibility, in both cases well after efficacy outcomes. Such delays reduce the clinical relevance of QoL findings and limit their integration into treatment decision-making at the time practice-changing evidence first emerges. A retrospective cohort study showed that, although half of trials specified QoL outcomes, only 20% reported them even after more than a decade, consistent with our results [44]. Because gains in PFS do not necessarily translate into improved HRQoL or delayed deterioration, and because PROs themselves have prognostic value (beyond clinical and disease-related factors), QoL results should accompany efficacy outcomes [45,46,47,48,49,50]. Hence, although efficacy remains the primary benchmark for regulatory approval, the increasing reliance on surrogate endpoints underscores the need for a more comprehensive evaluation that includes robust, timely, and meaningful QoL evidence.

Similar observations have been reported in other clinical settings and cancers such as lung cancer and colorectal cancer [51,52,53,54,55]. To enhance the quality and consistency of PRO data in clinical research, future trials should systematically apply established methodological frameworks throughout the research lifecycle. SPIRIT-PRO (2018) should guide the inclusion of PROs in study protocols, CONSORT-PRO (2013) should inform transparent and complete reporting, and SISAQOL (2020) should support standardized analysis [10,15,16,56,57]. Although we acknowledge the length of protocol development, most recent trials were initiated after the publication of these guidelines, yet adherence remains suboptimal. As our analysis of CONSORT-PRO adherence demonstrates, there remains substantial room for improvement in aligning trial practices with these standards. In addition to these guidelines, the ESMO-MCBS QoL checklist provides a structured framework to ensure that QoL and PRO data are systematically and transparently evaluated when assessing the clinical benefit of new cancer therapies within the ESMO Magnitude of Clinical Benefit Scale (MCBS) [58]. Future trials should actively align with these and other established frameworks. Regulatory agencies, reimbursement bodies, and scientific journals have a key role in endorsing and enforcing these standards [43].

### Limitations of the Review

The ClinicalTrials.gov database does not comprehensively capture historical endometrial cancer trials, particularly chemotherapy studies conducted before or around the early 2000s, a period in which QoL or PRO measures were rarely incorporated into trial protocols. In addition, our analysis was limited to trials that included QoL endpoints, which may have reduced our ability to illustrate the broader issue of insufficient QoL reporting across clinical studies. Finally, we excluded phase II trials, as QoL and PRO endpoints typically fall outside their scope and are often not assessed in early-phase studies.

Several important questions fall outside the scope of this review but warrant further consideration. These include whether available QoL instruments adequately capture the experiences of patients receiving innovative treatments such as targeted therapies and immunotherapies, whether they are sufficiently sensitive to detect subtle yet clinically meaningful, and to define minimal important differences [40,59,60,61]. In addition, the degree to which patients are involved in trial design, particularly in selecting outcomes and QoL measures, remains an important area in need of greater attention [40,43]. Although the integration of PROs and QoL assessments into routine cancer care is an important topic, detailed consideration of implementation aspects lies beyond the scope of this review.

## 5. Conclusions

In conclusion, QoL must be elevated from a secondary consideration to an integral endpoint in advanced endometrial cancer trials, with prespecified, rigorous, PRO methodology and timely, transparent reporting aligned with established QoL and PRO frameworks.

## Figures and Tables

**Table 1:** Characteristics of the studies.

Trial, Author and Year of Primary Publication	Experimental Treatment Regimen	Schedule and Duration of Treatment *	Primary Endpoint	QoL Endpoint	QoL Data Available in Primary Publication
**First-line treatment advanced endometrial cancer**					
GOG0209 Miller D. 2020 [6]	Carboplatin and Paclitaxel	7 × CT q3w	OS	Secondary	yes
NRG-GY018 Eskander R. 2023 [19]	Pembrolizumab plus Chemotherapy	6 × CT-Pembrolizumab q3w → 14 × Pembrolizumab q6w	PFS	Secondary	no (QoL/PRO analyses ongoing)
RUBY part 1 Mirza M.R. 2023 [20]	Dostarlimab plus Chemotherapy	6 × CT-Dostarlimab q3w → Dostarlimab q6w up to 3y	OS (co-primary)	Secondary	yes
DUO-E Westin S. 2023 [21]	Durvalumab Plus Chemotherapy Followed by Maintenance Durvalumab with or Without Olaparib	6 × CT-Durvalumab q3w → Durvalumab q4w +/− Olaparib until progression	PFS	Secondary	no (QoL/PRO analyses ongoing)
AtTEnd Colombo N. 2024 [22]	Atezolizumab plus Chemotherapy	6–8 × CT-Atezolizumab q3w → Atezolizumab q3w until progression	OS (co-primary)	Secondary	yes
LEAP-001 Marth C. 2024 [23]	Lenvatinib Plus Pembrolizumab	35 × Pembrolizumab q3w + Lenvatinib until progression	OS (co-primary)	Secondary	yes
Maintenance therapy after first-line treatment					
SIENDO Vergote I. 2023 [24]	Oral Selinexor	Selinexor until progression	PFS	Secondary	yes
Second-line treatment advanced endometrial cancer					
KEYNOTE-775 Makker V. 2022 [25]	Lenvatinib plus Pembrolizumab	35 × Pembrolizumab q3w + Lenvatinib until progression	OS (co-primary)	Secondary	yes

* Treatment should be discontinued earlier in case of unacceptable toxicity or progressive disease; Abbreviations: CT, carboplatin-paclitaxel. q3w, every 3 weeks. OS, overall survival. PFS, progression-free survival. QoL, quality of life. PRO, patient-reported outcome.

**Table 2:** Per protocol planned HRQoL analyses versus reported HRQoL analyses in primary publication.

Trial	Per Protocol Planned Analyses	Reported Analyses in Primary Publication
**Mean scores at diferent time points**		
GOG0209 [6]	FACT-PWB + FWB, FACT-En-TOI, FACT/GOG-Ntx 4	FACT-PWB + FWB, FACT-En-TOI, FACT/GOG-Ntx 4
NRG-GY018 [19]	FACT-En TOI, PROMIS-Fatigue/-Physical function, FACT/GOG-Ntx4	Analyses ongoing
**Mean change from baseline**		
RUBY [20]	EORTC QLQ C30 GHS/QoL and QLQ-EN24 score and domain scores	EORTC-QLQ-C30 GHS/QoL scores
DUO-E [21]	EORTC QLQ C30 GHS/QoL score and domain scores, QLQ-EN24 and domain scores	Analyses ongoing
AtTEnd [22]	EORTC QLQ C30 GHS/QoL score and domain scores, QLQ-EN24 and domain scores	EORTC-QLQ-C30 GHS/QoL score
LEAP-001 [23]	EORTC QLQ-C30 GHS/QoL score and domain scores	EORTC QLQ-C30 and QLQ-EN24 scales, including GHS/QoL, functional, and symptom scales
SIENDO [24]	EORTC QLQ C30 GHS/QoL score and domain scores, QLQ-EN24 and domain scores	EORTC QLQ-C30 GHS/QoL score
KEYNOTE-775 [25]	EORTC QLQ C30 GHS/QoL score and domain scores, QLQ-EN24 and domain scores	EORTC QLQ-C30 GHS/QoL score
**Time to deterioration**		
DUO-E [21]	Role and physical functioning score of the EORTC QLQ-C30 and back/pelvic pain, urological symptom and GI symptom subscales of the QLQ-EN24	Analyses ongoing
KEYNOTE-775 [25]	EORTC QLQ C30 GHS/QoL score and domain scores, QLQ-EN24 and domain scores	Not mentioned
**Response proportion**		
AtTEnd [22]	FACT-item GP5	Not mentioned

Abbreviations: See abbreviation list at the end.

**Table 3:** HRQoL and PRO measurement tools.

	GOG0209 [6]	NRG-GY018 [19]	RUBY [20]	DUO-E [21]	AtTEnd [22]	LEAP-001 [23]	SIENDO [24]	KEYNOTE-775 [25]
EORTC QLQ-C30			X	X	X	X	X	X
EORTC QLQ-EN24			X	X	X	X	X	X
EQ-5D-5L			X	X	X	X	X	X
FACT-En TOI	X	X						
FACT/GOG-Neurotoxicity 4	X	X						
FACT-item GP5		X			X			
FACT-PWB/-FWB	X							
PROMIS-Fatigue/-Physical function		X						
PRO-CTCAE				X				
PGIS, PGIC, PGI-TT, PGI-BR				X				

Abbreviations: See abbreviation list at the end.

**Table 4:** HRQoL/PRO measurement tools, frequency and duration of follow-up.

Trial	PRO Instrument(s)	Frequency of Measurements and Duration of Follow-Up
GOG0209 [6]	FACT-PWB/-FWB FACT-En-TOI FACT/GOG-Ntx-4	At 6, 15, and 26 weeks
NRG-GY018 [19]	FACT-En-TOI PROMIS-Fatigue PROMIS-Physical function FACT/GOG-Ntx-4 FACT-item GP5	At 6, 15, 24 and 51–54 weeks
RUBY [20]	EORTC QLQ-C30 EORTC QLQ-EN24 EQ-5D-5L	At every treatment and every 90 days during survival follow up period (up to 4 years after enrollment of last subject)
DUO-E [21]	EORTC QLQ-C30 EORTC QLQ-EN24 EQ-5D-5L PRO-CTCAE PGIS, PGIC, PGI-TT, PGI-BR	Every 3 weeks until week 18, and then every 4 weeks until second progression
AtTEnd [22]	EORTC QLQ-C30 EORTC QLQ-EN24 EQ-5D-5L FACT-item GP5	At cycles 3 and 6 and thereafter every 12 weeks until second progression or one-year of follow-up (whichever comes first)
LEAP-001 [23]	EORTC QLQ-C30 EORTC-QLQ-EN24 EQ-5D-5L	Every 3 weeks until cycle 35, then every 4 weeks until cycle 60
SIENDO [24]	EORTC QLQ-C30 EORTC QLQ-EN24 EQ-5D-5L	Every 12 weeks during the study period, at progression of disease and post progression at 3 and 6 months
KEYNOTE-775 [25]	EORTC QLQ-C30 EORTC QLQ-EN24 EQ-5D-5L	Every 3 to 4 weeks during treatment (depending on treatment arm) and thereafter for the equivalent of 4 cycle lengths

Abbreviations: See abbreviation list at the end.

**Table 5:** Compliance with CONSORT-PRO Extension Criteria *.

	GOG0209 [6]	NRG-GY018 [19]	RUBY [20]	DUO-E [21]	AtTEnd [22]	LEAP-001 [23]	SIENDO [24]	KEYNOTE-775 [25]
The PRO should be identified in the abstract as a primary or secondary outcome (1)	X	-	-	-	-	-	-	-
The PRO hypothesis should be stated and relevant domains identified, if applicable (2)	x	x	-	-	-	-	-	-
Evidence of PRO instrument validity (3a)		X	-	x	-	x	-	x
Methods of data collection (paper, telephone, electronic, other) (3b)	x	x	-	x	x	x	-	x
Statistical approaches for dealing with missing data are explicitly stated (4)		x	x	x	x	X	X	x
PRO–specific limitations and implications for generalizability and clinical practice (5)	X	-	-	-	-	-	-	-

* a green “X” indicates compliance in the primary report; a black “x” indicates that the criterion was included in the protocol but not reported in the primary publication (not applicable to item (1) ); a “-“ indicates that the criterion was neither addressed in the protocol nor in the primary report.

**Table 6:** Reporting timeline of efficacy outcomes and quality of life results in clinical trials (including journal, impact factor and congress presentation modality).

Trial	Primary Results		Secondary QoL/PRO Results	
	Congress Presentation	Primary Publication *	Congress Presentation QoL	Secondary Publication QoL *
GOG0209		September 2020 [6] JCO (IF 42.1)		
NRG-GY018	March 2023 [26] SGO (oral session)	March 2023 [19] NEJM (IF 96.3)	March 2025 [27] SGO (oral session)	
RUBY	March 2023 [28] SGO (oral session)	March 2023 [20] NEJM (IF 96.3)	June 2023 [29] ASCO (oral session)	April 2025 [30] IJGC (IF 4.7)
DUO-E	October 2023 [31] ESMO (oral session)	October 2023 [21] JCO (IF 42.1)	February 2025 [32] ESGO (oral session)	
AtTEnd	October 2023 [33] ESMO (oral session)	August 2024 [22] Lancet Oncology (IF 35.9)	June 2024 [34] ESMO-GC (oral session)	
LEAP-001	March 2024 [35] SGO (oral session)	November 2024 [23] JCO (IF 43.4)		
SIENDO	March 2022 [36] SGO (oral session)	September 2023 [24] JCO (IF 42.1)		
KEYNOTE-775	March 2021 [37] SGO (oral session)	January 2022 [25] NEJM (IF 158.5)	June 2021 [38] ASCO (poster session)	March 2023 [39] EJC (IF 7.6)

* Impact factors (IF) reported correspond to the year of publication or, for studies published in 2025, to the most recent available impact factor (e.g., 2024).

## Data Availability

The original contributions presented in this study are included in the article. Further inquiries can be directed to the corresponding author.

## Supplementary Materials

The following supporting information can be downloaded at https://www.mdpi.com/article/10.3390/cancers18020258/s1: Table S1: PRISMA_2020_checklist. Reference [62] is cited in Supplementary Materials.

## Abbreviations

The following abbreviations are used in this manuscript:

HRQoL	Health-related quality of life
PROs	Patient-reported outcomes
RCTs	Randomized controlled trials
CONSORT-PRO	Consolidated Standards of Reporting Trials—Patient-Reported Outcomes (Extension)
EORTC	European Organisation for Research and Treatment of Cancer
EORTC QLQ-C30	European Organisation for Research and Treatment of Cancer Quality of Life Questionnaire–Core 30
EORTC QLQ-EN24	European Organisation for Research and Treatment of Cancer Quality of Life Questionnaire–Endometrial Cancer Module
EQ-5D-5L	EuroQol 5-Dimension 5-Level Questionnaire
OS	Overall survival
TCGA	The Cancer Genome Atlas
PFS	Progression-free survival
PARP	Poly(ADP-ribose) polymerase
QoL	Quality of life
SPIRIT-PRO	Standard Protocol Items: Recommendations for Interventional Trials—Patient-Reported Outcomes Extension
PRISMA	Preferred Reporting Items for Systematic reviews and Meta-Analyses
ESMO	European Society for Medical Oncology
ASCO	American Society of Clinical Oncology
SGO	Society of Gynecologic Oncology
ESGO	European Society of Gynecological Oncology
IGCS	International Gynecologic Cancer Society
PROSPERO	International Prospective Register of Systematic Reviews
SPSS	Statistical Package for the Social Sciences
CT	Carboplatin-Paclitaxel
Q3W	Every 3 weeks
FACT	Functional Assessment of Cancer Therapy
FACT-PWB	Physical Well-Being
FACT-FWB	Functional Well-Being
FACT-En TOI	Endometrial Trial Outcome
FACT/GOG-Ntx4	Gynecologic Oncology Group—Neurotoxicity 4-item scale
PROMIS	Patient-Reported Outcomes Measurement Information System
GHS/QoL	Global Health Status/Quality of Life (the mean of Q29 and Q30 of the EORTC QLQ-C30 questionnaire)
FACT/item GP5	“I am bothered by side effects of treatment”
PGIS	Patient Global Impression of Severity
PRO-CTCAE	Patient-Reported Outcomes version of the Common Terminology Criteria for Adverse Events
PGIC	Patient Global Impression of Change
PGI-TT	Patient Global Impression of Targeted Therapy
PGI-BR	Patient Global Impression of Benefit–Risk
IF	Impact factor
CSO	Common Sense Oncology
SISAQOL	Setting International Standards in Analyses of Patient-Reported Outcomes and Quality of Life Endpoints in Cancer Trials
ESMO-MCBS	European Society for Medical Oncology Magnitude of Clinical Benefit Scale
